# Supplementary material for: Educational level and alcohol use in adolescence and early adulthood—The role of social causation and health-related selection—The TRAILS Study
Source: PLoS One. 2022 Jan 19;17(1):e0261606. doi: 10.1371/journal.pone.0261606 (PMC8769339; doi:10.1371/journal.pone.0261606)

S2 Fig. Path diagram of a cross-lagged panel model adjusted for time-invariant baseline characteristics.

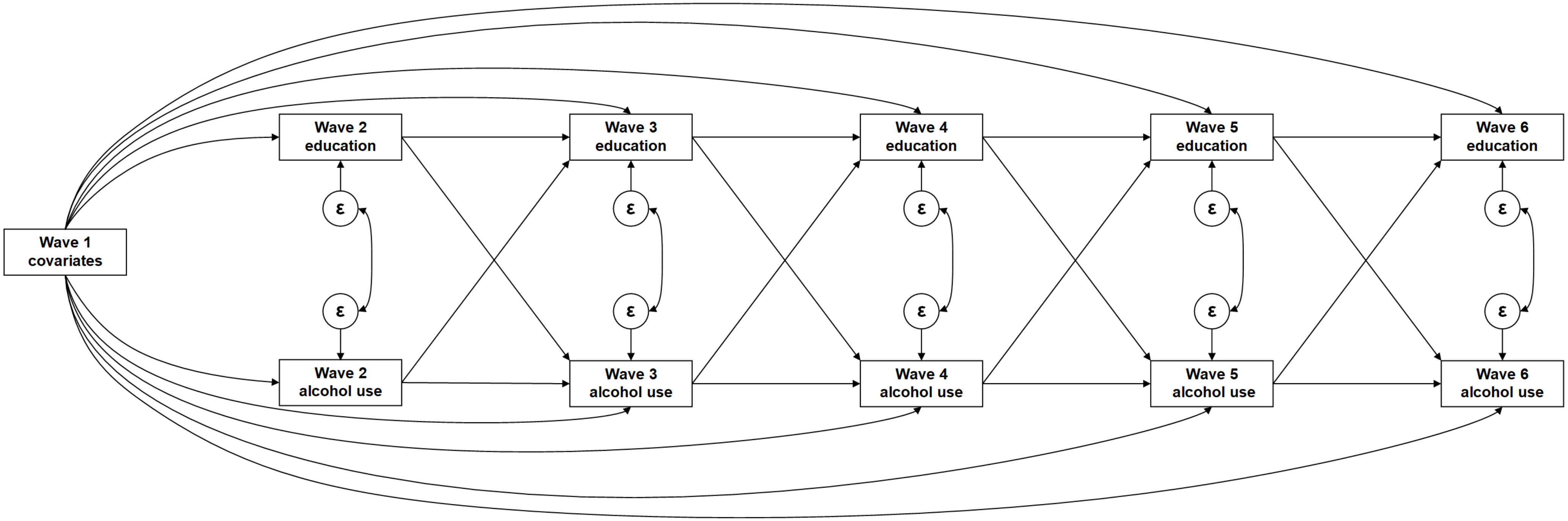

Supplement: S2 Fig — (PDF) [file pone.0261606.s002.pdf]
