# Supplementary material for: Educational level and alcohol use in adolescence and early adulthood—The role of social causation and health-related selection—The TRAILS Study
Source: PLoS One. 2022 Jan 19;17(1):e0261606. doi: 10.1371/journal.pone.0261606 (PMC8769339; doi:10.1371/journal.pone.0261606)

S3 Fig. Path diagrams of one-sided cross-lagged panel models with fixed effects according to the specification by Allison et al. [26]; separate fixed effects models were fit to assess each of the two hypothesized causal directions between educational level and alcohol use.

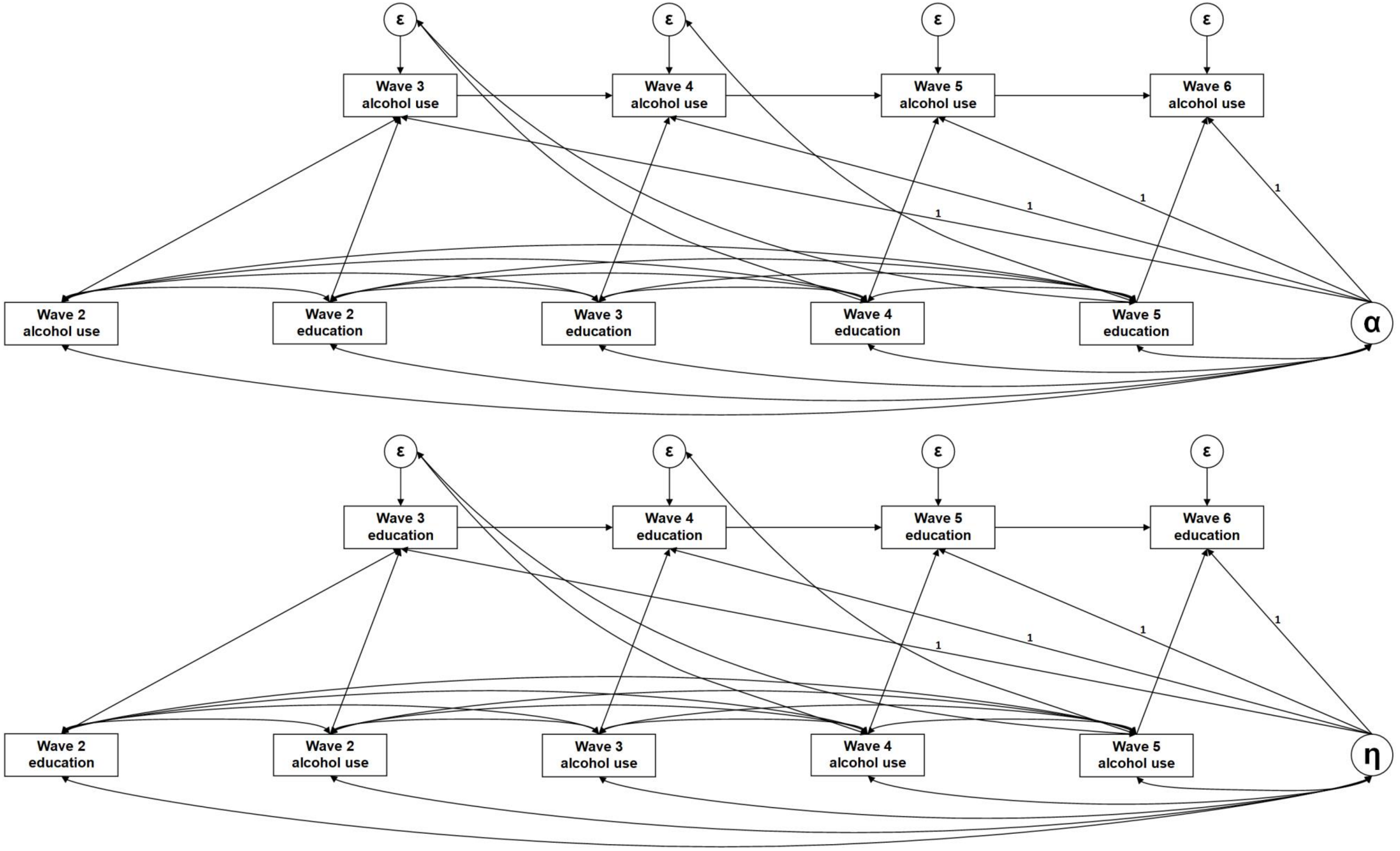

Supplement: S3 Fig — (PDF) [file pone.0261606.s003.pdf]
