# Supplementary material for: Educational level and alcohol use in adolescence and early adulthood—The role of social causation and health-related selection—The TRAILS Study
Source: PLoS One. 2022 Jan 19;17(1):e0261606. doi: 10.1371/journal.pone.0261606 (PMC8769339; doi:10.1371/journal.pone.0261606)
Supplement: S4 Fig — Model 1: bivariate cross-lagged panel model. Model 2: cross-lagged panel model adjusted for demographics (age, gender, area of residence, and ethnicity). Model 3: cross-lagged panel model adjusted for demographics, and parental socioeconomic status. Model 4: cross-lagged panel model adjusted for demographics, parental socioeconomic status, and adolescent psychological characteristics (IQ, effortful control). Edu = educational level; Alc = alcohol use. Boldface denotes statistical significance at p < 0.05. (PDF) [file pone.0261606.s004.pdf]

S4 Fig. Bidirectional associations between educational level and alcohol use in the TRAILS Study (the Netherlands, 2000–2017, N = 2,229); sequentially adjusted linear regression coefficients (stdyx-standardized ß-coefficient, robust standard error, p-value) for different sets of covariates.

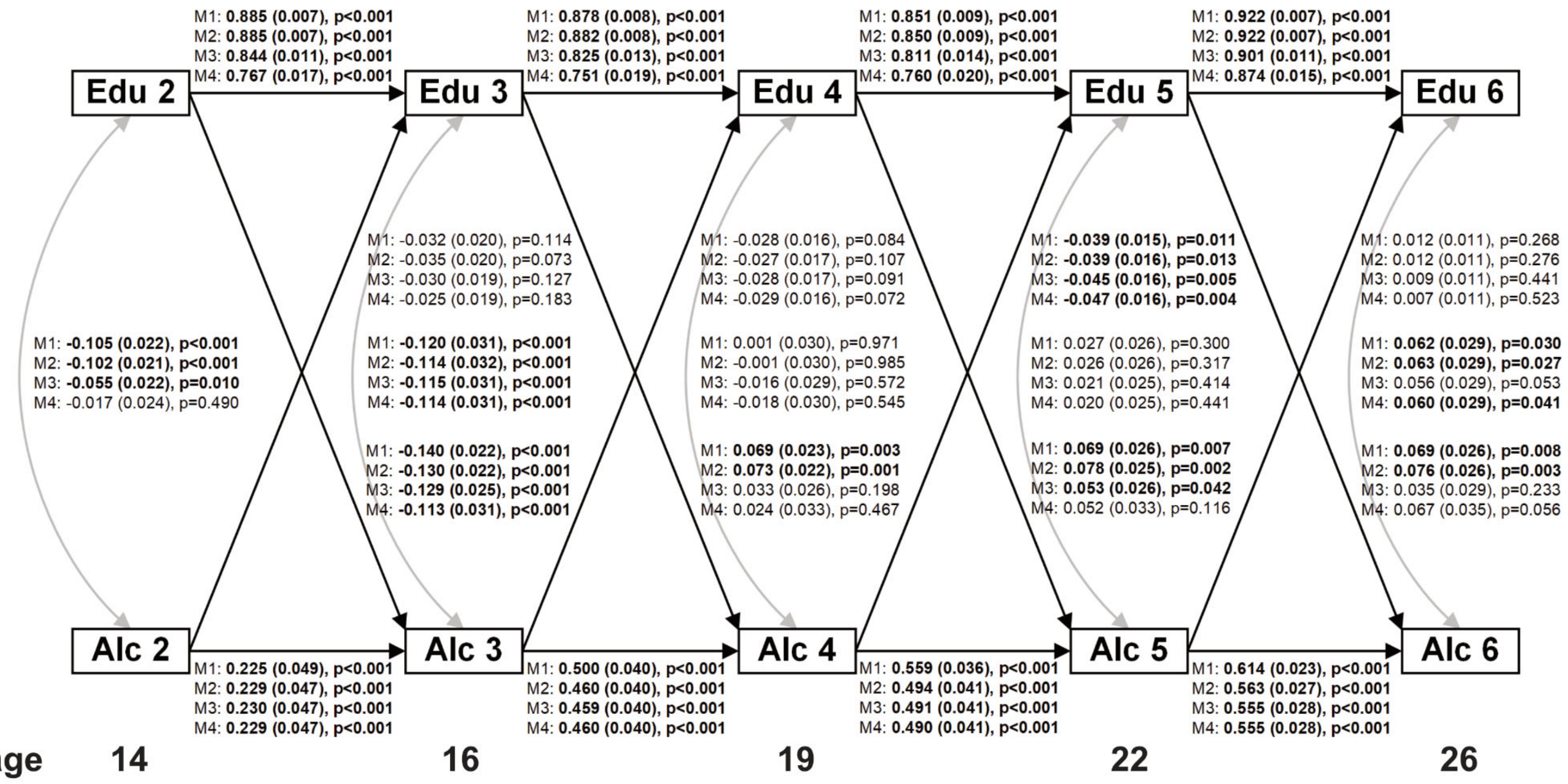

	CFI	TLI	SRMR	RMSEA
**Model 1**	0.984	0.969	0.032	0.045
**Model 2**	0.990	0.948	0.019	0.044
**Model 3**	0.992	0.956	0.017	0.039
**Model 4**	0.994	0.967	0.014	0.033

Model 1: bivariate cross-lagged panel model.
Model 2: cross-lagged panel model adjusted for demographics (age, gender, area of residence, and ethnicity).
Model 3: cross-lagged panel model adjusted for demographics, and parental socioeconomic status.
Model 4: cross-lagged panel model adjusted for demographics, parental socioeconomic status, and adolescent psychological characteristics (IQ, effortful control).
Edu = educational level; Alc = alcohol use.
Boldface denotes statistical significance at $p < 0.05$.
