# Supplementary material for: Educational level and alcohol use in adolescence and early adulthood—The role of social causation and health-related selection—The TRAILS Study
Source: PLoS One. 2022 Jan 19;17(1):e0261606. doi: 10.1371/journal.pone.0261606 (PMC8769339; doi:10.1371/journal.pone.0261606)
Supplement: S5 Fig — Model 1: bivariate cross-lagged panel model. Model 2: cross-lagged panel model adjusted for age, gender, area of residence, ethnicity, parental socioeconomic status, IQ, and effortful control at baseline (wave 1). Model 3: cross-lagged panel models with fixed effects–adjustment for time-invariant characteristics was performed by inclusion of a latent variable. Edu = educational level; Alc = alcohol use. Boldface denotes statistical significance at p < 0.05. (PDF) [file pone.0261606.s005.pdf]

**S5 Fig. Bidirectional associations between educational level and alcohol use in the TRAILS Study (the Netherlands, 2000–2017, N = 2,229); linear regression coefficients (stdyx-standardized ß-coefficient, robust standard error, p-value) from cross-lagged panel models without (Model 1 and 2) and with fixed effects (Model 3); the binge drinking item was removed from the AUDIT-C in these models.**

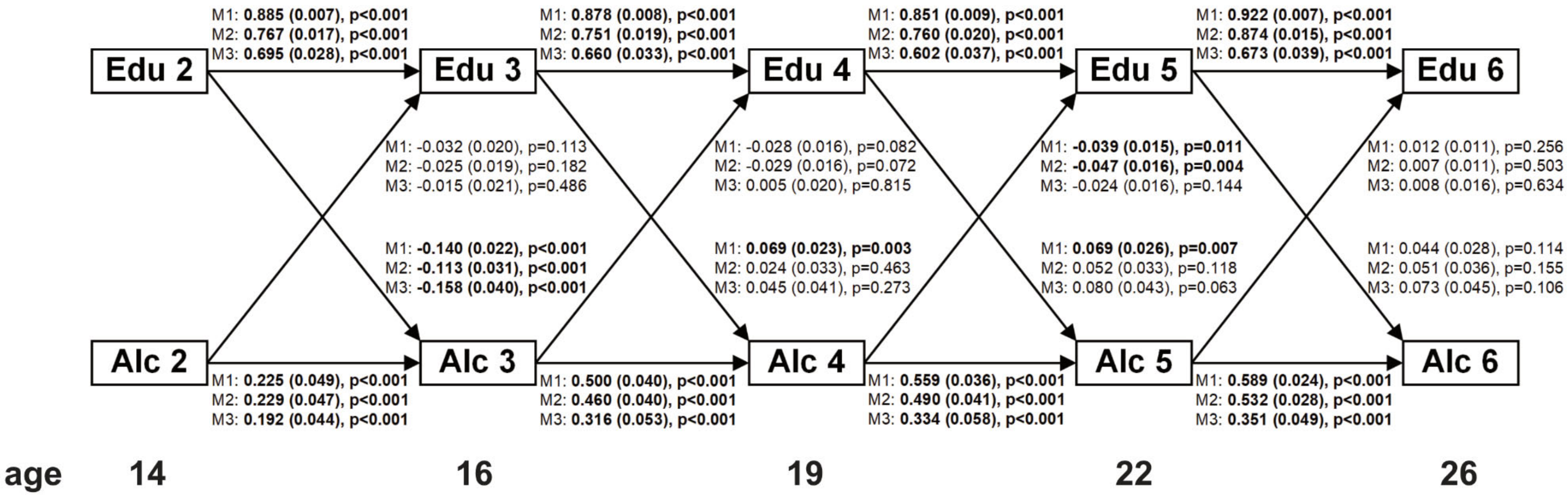


| | CFI | TLI | SRMR | RMSEA |
|---|---|---|---|---|
| **Model 1** | 0.985 | 0.972 | 0.030 | 0.043 |
| **Model 2** | 0.995 | 0.972 | 0.013 | 0.030 |
| **Model 3** | | | | |
| ***a) Education → Alcohol use*** | 1.000 | 0.998 | 0.008 | 0.011 |
| ***b) Alcohol use → Education*** | 0.997 | 0.989 | 0.007 | 0.029 |

Model 1: bivariate cross-lagged panel model.
Model 2: cross-lagged panel model adjusted for age, gender, area of residence, ethnicity, parental socioeconomic status, IQ, and effortful control at baseline (wave 1).
Model 3: cross-lagged panel models with fixed effects – adjustment for time-invariant characteristics was performed by inclusion of a latent variable.
Edu = educational level; Alc = alcohol use.
**Boldface** denotes statistical significance at $p < 0.05$.
