# Supplementary material for: Educational level and alcohol use in adolescence and early adulthood—The role of social causation and health-related selection—The TRAILS Study
Source: PLoS One. 2022 Jan 19;17(1):e0261606. doi: 10.1371/journal.pone.0261606 (PMC8769339; doi:10.1371/journal.pone.0261606)
Supplement: S6 Fig — Model 1: bivariate cross-lagged panel model. Model 2: cross-lagged panel model adjusted for age, gender, area of residence, ethnicity, parental socioeconomic status, IQ, and effortful control at baseline (wave 1). Model 3: cross-lagged panel models with fixed effects–adjustment for time-invariant characteristics was performed by inclusion of a latent variable. Edu = educational level; Alc = alcohol use. Boldface denotes statistical significance at p < 0.05. (PDF) [file pone.0261606.s006.pdf]

**S6 Fig. Bidirectional associations between educational level and alcohol use in the TRAILS Study (the Netherlands, 2000–2017, N = 2,229); regression coefficients (stdyx-standardized ß-coefficient, robust standard error, p-value) from cross-lagged panel models without (Model 1 and 2) and with fixed effects (Model 3), using the Bayes estimator; educational level from wave 3 to wave 5 was declared as "categorical" in Mplus.**

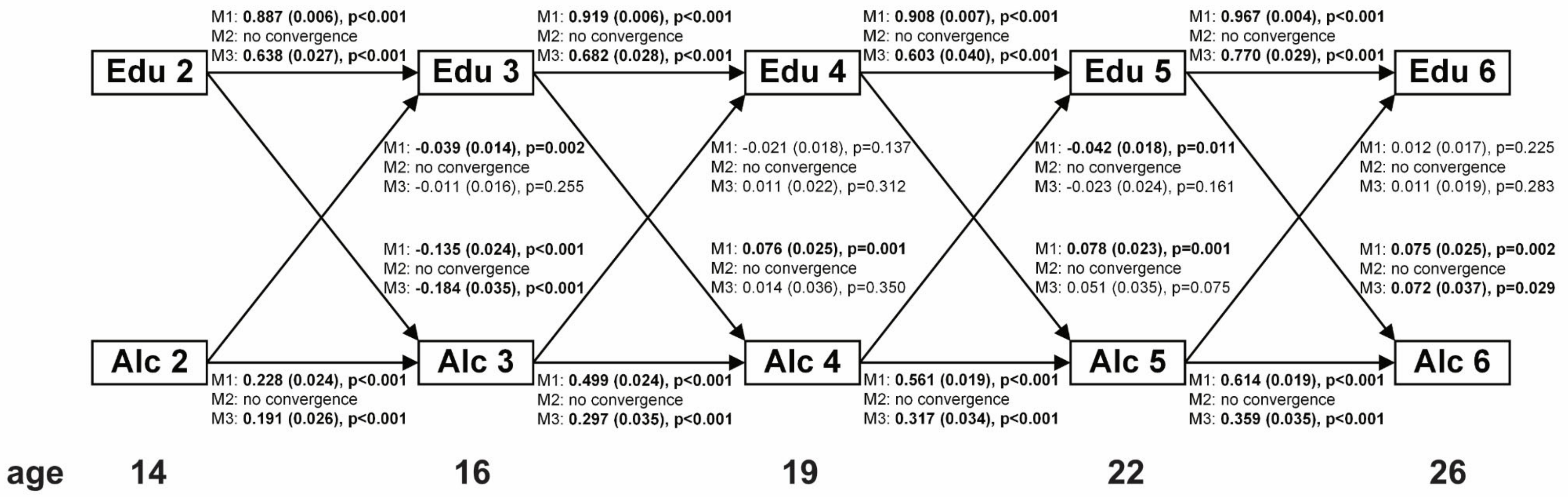


| | Posterior predictive p-value | 95% confidence interval for the difference between the observed and the replicated chi-square values |
|---|---|---|
| **Model 1** | 0.229 | -21.324 – 44.649 |
| **Model 2** | no convergence | no convergence |
| **Model 3** | | |
| ***a) Education → Alcohol use*** | 0.398 | -26.475 – 32.540 |
| ***b) Alcohol use → Education*** | 0.433 | -27.114 – 31.927 |

Model 1: bivariate cross-lagged panel model.
Model 2: cross-lagged panel model adjusted for age, gender, area of residence, ethnicity, parental socioeconomic status, IQ, and effortful control at baseline (wave 1).
Model 3: cross-lagged panel models with fixed effects – adjustment for time-invariant characteristics was performed by inclusion of a latent variable.
Edu = educational level; Alc = alcohol use.
**Boldface** denotes statistical significance at $p < 0.05$.
