# Supplementary material for: Educational level and alcohol use in adolescence and early adulthood—The role of social causation and health-related selection—The TRAILS Study
Source: PLoS One. 2022 Jan 19;17(1):e0261606. doi: 10.1371/journal.pone.0261606 (PMC8769339; doi:10.1371/journal.pone.0261606)
Supplement: S1 Table — SD = standard deviation. P-values were computed using chi-squared tests for categorical variables and two-sample t-tests for continuous variables. (PDF) [file pone.0261606.s007.pdf]

S1 Table. Attrition analysis – characteristics of young adults remaining in the TRAILS Study (the Netherlands, 2000–2017, N = 2,229) at wave 6, compared to participants who had dropped out of the cohort between wave 2 and wave 5.

	**Participants remaining in TRAILS by wave 6**		**Drop-outs**		**P-value**
**N participants, %**	1,616	(72.50)	613	(27.50)	
**Baseline characteristics**					
**Male gender, N (%)**	735	(45.48)	363	(59.22)	<0.001
**Non-Dutch ethnicity, N (%)**	155	(9.59)	146	(23.82)	<0.001
**Age, mean (SD)**	11.09	(0.56)	11.16	(0.54)	0.006
**Parental socioeconomic status (SES), mean (SD)**	0.10	(0.76)	-0.44	(0.77)	<0.001
**Wechsler Intelligence Deviation Quotient (IQ), mean (SD)**	99.64	(14.54)	90.67	(14.24)	<0.001
**Effortful control, mean (SD)**	3.27	(0.68)	3.09	(0.67)	<0.001
**Educational level, mean (SD)**					
Wave 2	2.53	(1.14)	1.72	(0.97)	<0.001
Wave 3	2.64	(1.10)	1.95	(1.06)	<0.001
Wave 4	2.73	(0.95)	2.39	(1.02)	<0.001
Wave 5	2.83	(0.90)	2.29	(1.01)	<0.001
**Alcohol use (quantity-frequency score), mean (SD)**					
Wave 2	1.55	(4.61)	1.94	(4.37)	0.112
Wave 3	6.48	(8.76)	9.58	(12.87)	<0.001
Wave 4	10.20	(11.74)	10.02	(10.82)	0.841
Wave 5	10.09	(11.01)	11.95	(10.98)	0.153

SD = standard deviation.

P-values were computed using chi-squared tests for categorical variables and two-sample t-tests for continuous variables.
