# Supplementary material for: Educational level and alcohol use in adolescence and early adulthood—The role of social causation and health-related selection—The TRAILS Study
Source: PLoS One. 2022 Jan 19;17(1):e0261606. doi: 10.1371/journal.pone.0261606 (PMC8769339; doi:10.1371/journal.pone.0261606)
Supplement: S2 Table — SD = standard deviation. aAlcohol use was measured using a quantity-frequency score from wave 2 to wave 5, and using the AUDIT-C at wave 6. P-values were computed using chi-squared tests for categorical variables and two-sample t-tests for continuous variables. (PDF) [file pone.0261606.s008.pdf]

S2 Table. Characteristics of participants with classifiable educational level compared to those with missing/unclassifiable educational level from wave 2 to wave 6 in the TRAILS Study (the Netherlands, 2000–2017, N = 2,229).

	N participants per wave		Male gender		Non-Dutch ethnicity		Age at baseline		Parental socioeconomic status (SES)		Wechsler Intelligence Deviation Quotient (IQ)		Effortful control, mean		Concurrent alcohol use[a]	
	**N**	**(%)**	**N**	**(%)**	**N**	**(%)**	**Mean**	**(SD)**	**Mean**	**(SD)**	**Mean**	**(SD)**	**Mean**	**(SD)**	**Mean**	**(SD)**
**Wave 2**	2,148	(100)														
Education complete	1,927	(89.71)	924	(47.95)	237	(12.30)	11.11	(0.56)	-0.03	(0.80)	97.58	(15.02)	3.22	(0.69)	1.63	(4.63)
Education unclassifiable/missing	221	(10.29)	130	(58.82)	35	(15.84)	11.07	(0.53)	-0.07	(0.75)	96.01	(13.44)	3.27	(0.64)	1.76	(3.83)
P-value				0.002		0.134		0.344		0.531		0.138		0.315		0.703
**Wave 3**	1,818	(100)														
Education complete	1,529	(84.10)	704	(46.04)	175	(11.45)	11.09	(0.56)	0.08	(0.78)	99.75	(14.85)	3.26	(0.69)	6.59	(9.28)
Education unclassifiable/missing	289	(15.90)	163	(56.40)	36	(12.46)	11.19	(0.57)	-0.32	(0.78)	91.25	(13.22)	3.04	(0.63)	9.78	(11.12)
P-value				0.001		0.623		0.004		<0.001		<0.001		<0.001		<0.001
**Wave 4**	1,880	(100)														
Education complete	1,507	(80.16)	671	(44.53)	160	(10.62)	11.09	(0.57)	0.13	(0.76)	100.34	(14.52)	3.30	(0.68)	9.99	(11.23)
Education unclassifiable/missing	373	(19.84)	227	(60.86)	52	(13.94)	11.10	(0.52)	-0.33	(0.74)	91.58	(13.13)	3.02	(0.63)	11.33	(13.87)
P-value				<0.001		0.069		0.693		<0.001		<0.001		0.001		0.110
**Wave 5**	1,781	(100)														
Education complete	1,429	(80.24)	624	(43.67)	147	(10.29)	11.10	(0.56)	0.12	(0.76)	100.13	(14.68)	3.30	(0.68)	10.24	(11.12)
Education unclassifiable/missing	352	(19.76)	219	(62.22)	52	(14.77)	11.11	(0.55)	-0.23	(0.76)	93.78	(13.88)	3.07	(0.66)	9.51	(9.69)
P-value				<0.001		0.017		0.608		<0.001		<0.001		<0.001		0.492
**Wave 6**	1,616	(100)														
Education complete	1,192	(73.76)	474	(39.77)	106	(8.89)	11.09	(0.56)	0.17	(0.75)	100.82	(14.47)	3.32	(0.67)	4.55	(2.37)
Education unclassifiable/missing	424	(26.24)	261	(61.56)	49	(11.56)	11.10	(0.56)	-0.09	(0.76)	96.31	(14.22)	3.11	(0.68)	4.97	(2.77)
P-value				<0.001		0.110		0.630		<0.001		<0.001		<0.001		0.078

SD = standard deviation.

[a]Alcohol use was measured using a quantity-frequency score from wave 2 to wave 5, and using the AUDIT-C at wave 6.

P-values were computed using chi-squared tests for categorical variables and two-sample t-tests for continuous variables.
