# Supplementary material for: Educational level and alcohol use in adolescence and early adulthood—The role of social causation and health-related selection—The TRAILS Study
Source: PLoS One. 2022 Jan 19;17(1):e0261606. doi: 10.1371/journal.pone.0261606 (PMC8769339; doi:10.1371/journal.pone.0261606)
Supplement: S3 Table — All predictors are mutually adjusted. From wave 3 to wave 6, all predictors are additionally adjusted for educational level and alcohol use in the preceding wave. Boldface denotes statistical significance at p < 0.05. (PDF) [file pone.0261606.s009.pdf]

S3 Table. The association between baseline characteristics (wave 1) and alcohol use and educational level from wave 2 to wave 6 in the TRAILS Study (the Netherlands, 2000–2017, N = 2,229) in the multivariate-adjusted cross-lagged panel model (model 2) in Fig 2; linear regression coefficients (stdyx-standardized ß-coefficient, robust standard error, p-value); all predictors are mutually adjusted.

	**Wave 2**	**Wave 3**	**Wave 4**	**Wave 5**	**Wave 6**
	**Educational level**				
**Male gender**	-0.029 (0.015), p=0.060	-0.019 (0.012), p=0.107	0.003 (0.012), p=0.799	0.001 (0.014), p=0.935	0.002 (0.013), p=0.852
**District**					
City of Groningen	ref	ref	ref	ref	ref
Leeuwarden	**-0.037 (0.017), p=0.034**	**0.027 (0.013), p=0.040**	-0.014 (0.014), p=0.331	0.027 (0.015), p=0.075	-0.009 (0.012), p=0.454
Assen	**-0.039 (0.019), p=0.046**	**-0.043 (0.014), p=0.001**	-0.003 (0.015), p=0.852	-0.020 (0.017), p=0.237	0.000 (0.015), p=0.979
Other regions	**-0.057 (0.019), p=0.003**	-0.028 (0.015), p=0.059	0.018 (0.015), p=0.235	-0.010 (0.017), p=0.549	-0.011 (0.014), p=0.403
**Non-Dutch ethnicity**	0.009 (0.016), p=0.570	**0.033 (0.012), p=0.007**	**0.034 (0.014), p=0.014**	0.007 (0.017), p=0.668	**0.031 (0.013), p=0.018**
**Age**	0.001 (0.018), p=0.949	-0.015 (0.014), p=0.275	0.008 (0.014), p=0.566	**-0.033 (0.016), p=0.042**	-0.007 (0.013), p=0.613
**Parental SES**	**0.280 (0.017), p<0.001**	**0.081 (0.014), p<0.001**	**0.109 (0.015), p<0.001**	**0.073 (0.018), p<0.001**	**0.038 (0.014), p=0.007**
**IQ**	**0.462 (0.016), p<0.001**	**0.075 (0.015), p<0.001**	**0.081 (0.015), p<0.001**	**0.067 (0.018), p<0.001**	**0.039 (0.014), p=0.004**
**Effortful control**	**0.249 (0.017), p<0.001**	**0.079 (0.014), p<0.001**	**0.063 (0.015), p<0.001**	0.032 (0.017), p=0.055	0.021 (0.013), p=0.112
	**Alcohol use**				
	**Quantity-frequency score**				**AUDIT-C score**
**Male gender**	-0.024 (0.022), p=0.267	**0.174 (0.023), p<0.001**	**0.213 (0.023), p<0.001**	**0.215 (0.025), p<0.001**	**0.128 (0.027), p<0.001**
**District**					
City of Groningen	ref	ref	ref	ref	ref
Leeuwarden	0.004 (0.027), p=0.896	0.051 (0.027), p=0.057	-0.033 (0.026), p=0.208	-0.012 (0.023), p=0.610	-0.034 (0.026), p=0.197
Assen	**-0.052 (0.027), p=0.049**	-0.010 (0.025), p=0.697	-0.032 (0.025), p=0.204	-0.007 (0.025), p=0.772	-0.028 (0.028), p=0.330
Other regions	-0.008 (0.029), p=0.767	0.035 (0.032), p=0.286	-0.004 (0.025), p=0.861	-0.014 (0.026), p=0.584	-0.016 (0.028), p=0.577
**Non-Dutch ethnicity**	0.000 (0.030), p=0.995	**-0.058 (0.023), p=0.011**	**-0.044 (0.018), p=0.018**	-0.016 (0.023), p=0.494	-0.038 (0.032), p=0.243
**Age**	0.002 (0.029), p=0.956	0.030 (0.026), p=0.254	0.000 (0.025), p=0.994	-0.036 (0.026), p=0.173	-0.012 (0.027), p=0.665
**Parental SES**	**-0.086 (0.021), p<0.001**	0.000 (0.026), p=0.987	**0.075 (0.027), p=0.005**	0.044 (0.027), p=0.107	**0.085 (0.029), p=0.004**
**IQ**	**-0.052 (0.026), p=0.045**	-0.020 (0.032), p=0.531	0.030 (0.028), p=0.293	0.021 (0.027), p=0.430	-0.021 (0.028), p=0.452
**Effortful control**	-0.042 (0.024), p=0.079	-0.011 (0.027), p=0.682	-0.016 (0.025), p=0.535	-0.024 (0.024), p=0.323	-0.051 (0.027), p=0.056

All predictors are mutually adjusted. From wave 3 to wave 6, all predictors are additionally adjusted for educational level and alcohol use in the preceding wave.
Boldface denotes statistical significance at p < 0.05.
