# Supplementary material for: Educational level and alcohol use in adolescence and early adulthood—The role of social causation and health-related selection—The TRAILS Study
Source: PLoS One. 2022 Jan 19;17(1):e0261606. doi: 10.1371/journal.pone.0261606 (PMC8769339; doi:10.1371/journal.pone.0261606)
Supplement: S4 Table — All predictors are mutually adjusted. Boldface denotes statistical significance at p < 0.05. (PDF) [file pone.0261606.s010.pdf]

S4 Table. Post-hoc analysis – the association between baseline characteristics (wave 1) and alcohol use at wave 2 in the TRAILS Study (the Netherlands, 2000–2017, N = 2,229) in the multivariate-adjusted cross-lagged panel model (Fig 2, Model 2) after additionally regressing wave 2 alcohol use on wave 2 education; linear regression coefficients (stdyx-standardized ß-coefficient, robust standard error, p-value); all predictors are mutually adjusted.

	**Alcohol quantity-frequency score**
**Male gender**	-0.025 (0.022), p=0.254
**District**	
City of Groningen	ref
Leeuwarden	0.003 (0.027), p=0.921
Assen	**-0.053 (0.026), p=0.042**
Other regions	-0.010 (0.028), p=0.727
**Non-Dutch ethnicity**	0.000 (0.030), p=0.989
**Age**	0.002 (0.029), p=0.955
**Parental socioeconomic status (SES)**	**-0.079 (0.024), p=0.001**
**Wechsler Intelligence Deviation Quotient (IQ), mean (SD)**	-0.041 (0.034), p=0.222
**Effortful control**	-0.036 (0.027), p=0.177
**Wave 2 educational level**	-0.024 (0.035), p=0.490
**Fit measures**	
**CFI**	0.994
**TLI**	0.967
**SRMR**	0.014
**RMSEA**	0.033

All predictors are mutually adjusted.

Boldface denotes statistical significance at $p < 0.05$.
