# Supplementary material for: Educational level and alcohol use in adolescence and early adulthood—The role of social causation and health-related selection—The TRAILS Study
Source: PLoS One. 2022 Jan 19;17(1):e0261606. doi: 10.1371/journal.pone.0261606 (PMC8769339; doi:10.1371/journal.pone.0261606)
Supplement: Attachment — Submitted filename: response_to_reviewers.docx [file pone.0261606.s011.docx]

| **Reviewer 1** |  |
| --- | --- |
| Abstract |  |
| 1. You may want to include the number of waves in the methods section | We have now added information about waves to the methods section of the abstract. |
| Introduction/ Literature Review |  |
| 2. You may want to be more explicit about your research question in the current study section. You could also incorporate the theories you are leveraging if you wanted to add the direction of effects into your research questions. | We now explicitly state the research questions, as well as our expectations based on the literature (p. 7, 8). |
| Methods |  |
| 3. Can you include the omega reliability for EATQ-R and AUDIT-C rather than alpha?  Also, for the measures, can you include the number of items?  Also, binge drinking is defined differently for males and females, was this adjusted for in that item? It looks like six or more drinks were used for both males and females. | We were able to calculate MacDonald’s Omega for the EATQ-R effortful control scale, which was 0.869, which is very similar to the corresponding Cronbach’s alpha of 0.86. We were not able to achieve convergence for the models to estimate Omega for the AUDIT-C in both MPLUS and STATA. We would therefore prefer to report Cronbach’s alpha, which is the most used measure of internal consistency throughout the literature.  The number of items is given on p. 10 for the EATQ-R on p. 10 and pp. 8-9 for the AUDIT-C.  TRAILS used the same questionnaires for boys and girls, with a cut-off of 6 or more glasses to define binge drinking. We took into account potential gender differences by statistical adjustment, as we did in our traditional CLPMs, or using within-between separation (i.e. fixed effects). Furthermore, our conclusions did not differ substantially when the binge drinking item was removed (see **S5 Fig**). |
| 4. The methods section is clear. I appreciate the detailed analytic approach. Why wasn’t the CLPM with fixed effects used as the primary model (and remove the CLPM)?  The authors cite Hamaker’s work, so they are familiar with the issue of the traditional CLPM (convergence effect aka “smushed effect.” Presenting Allison’s approach or the RI-CLPM (or the ALT-SR model see Curran) as the primary model would be more appropriate, mainly since this is a longitudinal study. Partitioning variance at the within- and between-person levels seems more suited to examine changes across these developmental stages.  You could also conduct a Chi Bar test to test whether a model like the RI-CLPM would be warranted.  I think the RI-CLPM is a good approach because you can report both between-person intercept (across time or at each time point) as well as within-person lagged effects. A focus on this approach would provide mo re information, examine lagged effects at a more appropriate level of examining developmental changes, and addressing the significant issues and limitations of the CLPM (see Berry and Willoughby, 2017). | We thank the reviewer for this suggestion. Our work is inspired by Mackenbach (2012), who hypothesized that specific childhood characteristics may drive the association between health behaviours and socioeconomic status later in life. Therefore, visualizing the role of important childhood predictors of education and alcohol use (i.e. ‘indirect’ social causation/ health-related selection) in longitudinal associations was an important objective of our study.  However, this type of question cannot be addressed with Allison’s fixed effects model or the RI-CLPM, as these models simply “adjust away” the effect of all time-stable characteristics. In these models, it is no longer possible to, for example, distinguish between the effects of parental SES and IQ on cross-lagged paths, as we did in the sequentially adjusted traditional CLPMs in **S4 Fig.** Using our approach, we, for example, observed that the path from education around age 16 to alcohol use around age 19 is driven by differences in parental SES. Finally, the fixed effects CLPM (and the RI-CLPM) does not allow for time-varying effects of time-stable variables, which is exactly what we found when considering the association of parental SES with alcohol use over time (see **S3 Table**). A similar approach to ours of comparing Allison’s fixed effects model to the ‘traditional’ CLPM has been previously used by, for example Magnusson Hanson et al. (2017).  It was unfortunately not possible to conduct a chi-bar-square test to compare our models as they are not nested (see also **S1-S3 Figs**).  We fully agree with the reviewer that a RI-CLPM is better able to capture developmental changes on the between-person level. Indeed, a main motivation for residual-level models like the RI-CLPM is an easy interpretability of the individual effects, which is not the case for observation-level models like the FE-CLPM (Andersen, 2021).  Nevertheless, in this particular case we preferred the FE-CLPM because:  1.) The FE-CLPM ‘social causation’ model can handle the ordinal education variable using linear regression and maximum likelihood without problems.  The one-sided FE-CLPM, as shown in Allison et al. (2017), does not place any form assumptions on the time-varying “exposure” variable (i.e. education) by making it exogenous at all measurement occasions (Andersen, 2021).  2.) Residual-level models like the RI-CLPM place potentially strong assumptions on the initial conditions of the model, assuming that the process under investigation is stationary and has been going on for long enough to reach equilibrium (Andersen, 2020). If the process is nonstationary, has not yet reached equilibrium, or has been knocked out of equilibrium, the RI-CLPM can give incorrect results. Observation-level models like the FE-CLPM do not have this limitation. Given that alcohol use shows substantial growth over the course of adolescence, we cannot assume stationarity of the process under study (Andersen, 2021). |
| 5. I’m also wondering if you could present the Intra Class Correlations so readers could get a sense of how much variance in the variables are within- or between people. I was most interested in the ICC for educational level. Is there wave-to-wave fluctuation in education level, or do most people remain on the same track? | Information on the ICC of education and alcohol use has been added to the manuscript (p. 14).  The ICC for education was 0.820, which indicates that 82% of the variance across the five measurements of educational level was due to differences between persons. This finding is in line with the high stability in educational level we found in CLPMs. The ICC for alcohol use was 0.293, suggesting that a substantial proportion of variance in alcohol use represents within-person fluctuations over time. This is likely because alcohol use increases substantially (within person) in most adolescents between age 14 and 26. |
| 6. The authors state, “male gender, non-Dutch ethnicity lower educational level, IQ, and effortful control, as well as those from lower SES households, were more likely to drop out of the study.” Since FIML was used to account for missing data, you could also note that these variables were also included in the model, thereby adjusting for potential bias due to missing data on these variables.  Also, you may want to report what the actual attrition is by showing the sample size at each wave since the table is only reported in supplemental. | We have now included all baseline variables as auxiliary variables in all analyses, in case they were not already included as predictors.  We now give more detailed information on attrition by each wave (p. 12). The sample size at each wave is given in Table 2. |
| 7. Are you using standard cutoffs for the fit indices listed to assess model fit? For example, are you following Hu and Bentler, 1999? I understand there are issues with cutoffs, but sometimes it’s good to state what you are using to guide the model fit assessment. | We now cite Hu & Bentler (1999) to support our conclusions on model fit (p. 12). |
| 8. I think the most crucial point is how each of the variables was treated in the CLPM. I could see one arguing that alcohol use was treated as a continuous variable; however, treating educational level as a continuous variable is not appropriate. I suggest you define this variable as categorical in Mplus and use the WLSMV estimator with theta parameterization, which is a probit model. You could also choose to use marginal maximum likelihood with a logit or probit link so you could invoke FIML to address missing data; however, remember this approach does not have a baseline model and thus has no fit indices. You could use this as a sensitivity analysis for the WLSMV model. The primary point is that it is not appropriate to treat an ordered categorical variable as continuous as it appears been done in the current analysis. This holds true for the linear regression models and the CLPM. | Our multivariate CLPM (Fig 2, Model 2) does not achieve convergence when using WLSMV or Bayesian estimators for categorical data.  It is not possible to run the FE-CLPM or the traditional CLPM using the logit or probit link with MLR, as these methods do not allow for correlations with error terms in endogenous categorical variables, which need to be specified in both types of models.  We therefore decided to conduct linear regression models, whilst addressing potential violations using robust maximum likelihood (MLR) with sandwich estimator. Furthermore, using the one-sided estimation of the fixed effects CLPM made the “social causation” model robust to any violations stemming from the ordinal nature of the education variable by making this variable exogenous.  We conducted a sensitivity analysis by using the “Bayes” estimator whilst declaring the education variable as “categorical”, to assess whether the aforementioned approach was sufficient to address potential bias due to the ordinal nature of the education variable (see **S6 Fig**). We were able to conduct this sensitivity analysis for the bivariate traditional CLPM and both fixed effects models. We were not able to achieve convergence of the multivariate traditional CLPM, as stated earlier. Overall, the results strongly resembled those obtained from the MLR models, with the exception of the path from alcohol use around age 14 to lower education around age 16 in the bivariate traditional CLPM, which was significant in the categorical model. However, this path was absent in the categorical Bayesian fixed effects model, similar to the MLR model.  We decided to use categorical Bayesian models for our sensitivity analysis rather than WLSMV, as both the Bayesian methods and MLR can treat missing data under the missing at random (MAR) assumption, while WLSMV cannot (see p.8 in Asparouhov and Muthen (2010b) and p. 18 in Asparouhov and Muthen (2010a)). |
| 9. Can you add the age, gender, area of residence, and ethnicity at baseline in **Table 3**? And create a table for them for the CLPM? | We have added a table (**S3 Table**) visualizing the mutually adjusted associations of the baseline predictors with education and alcohol use in the multivariate adjusted cross-lagged panel model (Model 2) in Figure 2 to the appendix.  We now used the data from this table in **Table 3**, which includes age, gender, area of residence, and ethnicity at baseline. |
| Results |  |
| 10. You may want to try to incorporate your research questions in the result section. You could also include social causation and health selection in this section. | We have added a more detailed description of causation/selection to the results section (pp. 14, 15). |
| 11. In general, the results are organized well. You could incorporate standardized effects in the results section so readers can get a sense of the magnitude of the effects. | Standardized effect sizes are now reported in the text, in line with the reviewers’ suggestion. |
| 12. Table 2: I do not think a test like ANOVAs should be conducted for descriptive tables. There are so many tests you had to run (10 comparisons for each variable across time). Were there any adjustments made to account for all these tests? Also, is it possible to clarify the differences in the subscript (a, b, c)? | We have removed the significance tests for the descriptive statistics. Multiple testing can indeed be a problem and the descriptive statistics are not the main substantive result of our study. |
| 13. Table 3: This linear regression model seems redundant. These effects are included in Model 2 of the CLPM, and it is more appropriate to report these findings rather than fitting another model to report demographic predictors. Also, why aren’t the controls include in the table? Further, Model 3 Table 3 note states that SES is included, but no effect is presented. | We now use the output of Model 2 in Figure 2 to estimate the associations of the childhood predictors with education and alcohol use at wave 2, as suggested. See also **S3 Table**. We now also include all controls in the tables. |
| 14. Within time correlations (residual correlations) are not in the figure. Can you add them to a table? Maybe supplemental? | Residual correlations for the traditional CLPMs are now shown in **S4 Fig**.  The FE-CLPM does not produce any residual correlations, except for the one between the initial observations. The coefficient for this correlation (i.e. between wave 2 education and alcohol use is ß -0.107, SE 0.023, p<0.001 for both the causation and selection models. |
| 15. What do you make of the difference between the CLPM and the Fixed effects model? I would argue that it is essential to plausibly disaggregate within- and between-person effects. In my view, all longitudinal models are inherently multilevel because persons are context. As such, it is important to disaggregate these substantively different levels of variance. How one differs from their own mean or trajectory over time (within-person) is not the same thing as how one differs from someone else (between-person). Both are meaningful but carry different substantive meanings. Allison’s approach is good; however, in the current study, the between-person effects should be interpreted as well (they are meaningful). | The fixed effects model by Allison does not give interpretable between-person estimates (see Andersen, 2021). While this clearly is a disadvantage of the FE-CLPM compared to the RI-CLPM, we believe that the advantages of using the FE-CLPM outweigh the disadvantages in our case, as argued above.  Except for one path, we found similar results in the multivariate adjusted traditional and fixed effects CLPM. This suggests that most of the relevant “between-person” differences that could “explain away” cross-lagged associations have been adjusted for in the multivariate CLPM.  In other words, we have been able to identify the most important time-stable “between-person” confounders. |
| Discussion |  |
| 16. Other limitations include the heavy use of single-item indicators, which assume measurement invariance over time. Further, not addressing the issue of polysubstance use, which is very common. As such, any harm attributed to alcohol may be better explained by the use of multiple substances. | We have now discussed quantity-frequency measures in the limitations (pp. 18). We have also addressed the role of polysubstance use in the limitations (p. 18). |
| 17. I appreciate the discussion of the findings. Can you add more about the practical and policy implications of the current work? | Information on practical and policy implications has been added to the conclusion (pp. 22, 23). |
| 18. I suggest that you temper the use of the word causation. The data and models are correlational. In addition, several confounds were not included in these models, and the study does not address the issue of polysubstance use, which has shown to be very common across this age range. As such, it is unknown in this study if harms associated with alcohol use are being misappropriated. | We have reduced the use of the word “causation” and now rather talk about the social causation theory or hypothesis.  We now address the issue of poly-substance use in the limitations (p. 18). However, particularly in the earlier age groups this might only apply to a very small proportion of adolescents. Drug use is rather uncommon around age 14 in Dutch general population samples.  Importantly, we do note that we did not find much evidence of health-related selection attributable to alcohol use in the fixed effects models. It is, however, possible that an unassessed time-invariant variable related to poly-substance use might contribute to the selection path we found between around age 19 and 22. |
| 19. Is this project preregistered on OSF? If so, you could include the link. | No, the project is not preregistered. Plos One uses a separate procedure for registered reports. |
| 20. Were the scripts uploaded to GitHub? If so, could you make the repository public and share the link? | The scripts for our analyses were uploaded to Github under the following link: <https://github.com/hschmengler/Educational-level-and-alcohol-use-in-adolescence-and-early-adulthood---the-TRAILS-Study> |
| 21. It appears that a lot of post hoc analyses were run in this study. I encourage the authors to focus on the primary models and limit the number of fitted models. The more models you run, the higher the likelihood of capitalizing on chance. Remember, at a p-value of .05, one out of 20 models is wrong, given chance alone. | We have removed significance tests from Table 1 and 2, in line with the reviewer’s suggestions. The only post-hoc analysis part of this study is the one in **S4 Table**, which shows the consequences of adjusting for education for the associations between parental SES, IQ, and effortful control and alcohol use at wave 2. |

| **Reviewer 2** |  |
| --- | --- |
| 22. The study does not comply with the PLOS data policy: “Why do we not allow an author to be the only point of contact for fielding requests for access to restricted data?  When possible, we recommend authors deposit restricted data to a repository that allows for controlled data access. If this is not possible, directing data requests to a non-author institutional point of contact, such as a data access or ethics committee, helps guarantee long term stability and availability of data. Providing interested researchers with a durable point of contact ensures data will be accessible even if an author changes email addresses, institutions, or becomes unavailable to answer requests.” | We have now provided contact information of the data manager, as well as the ethics committee in the data availability statement. TRAILS is a consortium which is independent of the authors of this publication. For data access requests, the TRAILS consortium can be contacted directly, as indicated in the data availability statement. The statement is written in a way which is similar to a previous Plos One publication on TRAILS, which was published under the same conditions (Peeters et al., 2019). |
| 23. “Social causation” and “health-related selection” should both be defined in the first paragraph of the abstract. | A definition of social causation and health-related selection has been added to the abstract. |
| 24. Page two, line 62: Please remove reference to “causation” as the data is not from an experiment incorporating randomization. Please use “social causation theory” or “social causation hypothesis” throughout the manuscript to avoid confusion. | We now use “theory” or “hypothesis” when referring to social causation and health-related selection in order to reduce causal language. |
| 25. Page 3, line 78: it is more correct to say that youngsters can affect their own SES through education. | We have made the suggested change. |
| 26. No psychometric evidence is provided for the validity of the quantity-frequency measure. How well did it correlate with audit-c? Can the authors provide any other evidence that the measure is reliable and valid? | Quantity-frequency measures are frequently used to assess alcohol use in adolescents, including in major cross-national surveys, such as ESPAD (Müller et al., 2010).  McKenna et al. (2018) performed a systematic review on the psychometric properties of alcohol consumption measures in general population surveys. Quantity frequency measures demonstrated good/adequate test-retest reliability, convergent validity, criterion validity, and hypothesis validity. Overall, their results indicated that quantity-frequency measures showed superior performance in psychometric terms, as compared to other measures (e.g. short-form recall measures, graduated-frequency measures) (McKenna et al., 2018).  We were not able to find any study which determined the correlation of the quantity-frequency measure used from wave 2 to 5 in TRAILS with the AUDIT-C at the same measurement occasion. However, the Pearson correlation between the full AUDIT-C and the sum score of the first two questions of the AUDIT-C at wave 6 of our study is 0.96. The first two questions of the AUDIT-C essentially constitute a quantity-frequency measure, though this is not exactly the same as the one in the previous waves of TRAILS. |
| 27. Has the method used to classify adolescent educational level been used in a published study before? Is it considered a valid way to quantify educational level? | Educational track membership has been frequently used in the literature to quantify educational differences in adolescent health in selective educational systems. See for example: de Looze et al. (2013); Richter et al. (2013); Veldman et al. (2014).  In the Netherlands, it is common to use our scale (or similar), with four categories. See de Looze et al. (2013) for an example from the HBSC Study.  Studies in countries with comprehensive educational systems, such as the USA, the UK, or Finland, use similar measures when investigating educational differences in young adult health (behaviours), for example by comparing college students to young adults who have followed vocational education See for instance: Aaltonen et al. (2016); Jang et al. (2019); Latvala et al. (2014); Slutske (2005); Sweeting et al. (2016). |
| 28. I have not come across the approach taken for the analogous CLPM with fixed effects models. Can the authors provide another reference, aside from Allison, that has used this approach to give confidence in the validity of the approach? It is not clear what it adds over the analytic approaches. | Allison’s CLPM with fixed effect is used widely throughout the literature. While the model originally has been originally published already in 2005 for SAS (Allison, 2005), the 2017 paper we cite validates Allison’s model by comparing it to the Arrellano-Bond estimator, which is frequently used in econometrics. According to Google Scholar, Allison et al. (2017), which also describes the MPLUS implementation we used, has received 132 citations to date. The article which describes the STATA implementation (xtdpdml) has received 61 citations (Williams et al., 2018).  For a discussion of the advantages of the model by Allison over alternative approaches see our response to point 4 and Andersen (2021).  Below, we provide several articles using Allison’s approach:  Hides, L., Chan, G., Dawe, S., McKetin, R., Kavanagh, D. J., Young, R. M., Teesson, M., & Saunders, J. B. (2021). Direction of the relationship between methamphetamine use and positive psychotic symptoms in regular methamphetamine users: evidence from a prospective cohort study. The British Journal of Psychiatry, 219(1), 361-367.  Jindra, C., Li, C., Tsang, R. S. M., Bauermeister, S., & Gallacher, J.  (2020). Depression and memory  function – evidence from cross-  lagged panel models with unit  fixed effects in ELSA and HRS.  Psychological Medicine, 1-9.  Nyberg, A., Peristera, P.,  Westerlund, H., Johansson, G.,  & Hanson, L. L. M. (2016).  Does job promotion affect  men’s and women’s health  differently? Dynamic panel  models with fixed effects.  International journal of  epidemiology, 46(4), 1137-1146.  Wu, H. F. (2021). Social  determination, health selection or indirect  selection? Examining the causal directions  between socioeconomic status and  obesity in the Chinese adult population.  Social Science & Medicine, 269, 113564. |
| 29. The data were not missing completely at random (MCAR). While Full Information Maximum Likelihood (FIML) estimation is appropriate to account for this, it should be clear that this is only case for models that include auxiliary variables predictive of missingness, i.e. those that include gender, ethnicity, and IQ. Differences between these models (i.e., Model 3) and others may be due to bias in accounting for missing data. | We have now included auxiliary variables in our structural equation models. All variables included in the fully adjusted multivariate CLPM (Figure 2, Model 2) were included as auxiliary variables in all other models if they were not part of these models. |
| 30. The linear regressions reported in table 3 are not well justified. What exactly are they trying to demonstrate? If it was predictors of educational track, wasn’t this already determined at wave 1? These regressions are also prone to bias related to missing data. Unlike FIML with auxiliary variables, this bias is not adequately accounted for. I think these regressions should probably be removed unless a compelling rationale can be provided. | We now use the output of Model 2 in **Table 2** to complete **Table 3**, as stated in our responses to points 9 and 13. This model includes all baseline predictors, so the inclusion of auxiliary variables is no longer necessary in this case.  The purpose of **Table 3** is to determine predictors of selection into different educational tracks. This selection happens around age 11 – 12, between wave 1 and 2 in TRAILS. We have now clarified the timing of the selection in our methods section (page 9).  In our CLPMs we found very high stability in educational track membership over the course of adolescence and young adulthood. Therefore, most health-related selection (i.e. assignment of educational level based on health-related characteristics) happens during the transition from primary to secondary school. |

| **Editor** |  |
| --- | --- |
| 31. Please ensure that your manuscript meets PLOS ONE's style requirements, including those for file naming. The PLOS ONE style templates can be found at  <https://journals.plos.org/plosone/s/file?id=wjVg/PLOSOne_formatting_sample_main_body.pdf> and  <https://journals.plos.org/plosone/s/file?id=ba62/PLOSOne_formatting_sample_title_authors_affiliations.pdf> | We have adjusted the manuscript to meet the Plos One criteria. |
| 32. We note that the grant information you provided in the ‘Funding Information’ and ‘Financial Disclosure’ sections do not match.  When you resubmit, please ensure that you provide the correct grant numbers for the awards you received for your study in the ‘Funding Information’ section. | We have removed information on funding from the acknowledgements section. The corrected statement on funding information is now included in the cover letter, as requested below. |
| 33. Thank you for stating the following in the Acknowledgments Section of your manuscript:  This study is made possible by the Consortium on Individual Development (CID). CID is funded through the Gravitation program of the Dutch Ministry of Education, Culture, and Science and the Netherlands Organization for Scientific Research (NWO) (grant number 024.001.003). This research is part of the TRacking Adolescents’ Individual Lives Survey (TRAILS). Organizations participating in TRAILS include various departments of the University Medical Center and University of Groningen, the Erasmus University Medical Center Rotterdam, Utrecht University, the Radboud Medical Center Nijmegen, and the Parnassia Bavo group, all in the Netherlands. TRAILS has been financially supported by various grants from NWO, ZonMW, GB-MaGW, the Dutch Ministry of Justice, the European Science Foundation, BBMRI-NL, and the participating universities. The authors would like to thank the participants of the TRAILS Study and all staff involved in the management and execution of this project.  We note that you have provided funding information that is not currently declared in your Funding Statement. However, funding information should not appear in the Acknowledgments section or other areas of your manuscript. We will only publish funding information present in the Funding Statement section of the online submission form.  Please remove any funding-related text from the manuscript and let us know how you would like to update your Funding Statement. Currently, your Funding Statement reads as follows:  TRAILS has been financially supported by various grants from NWO, ZonMW, GB-MaGW, the Dutch Ministry of Justice, the European Science Foundation, BBMRI-NL, and the participating universities.  Please include your amended statements within your cover letter; we will change the online submission form on your behalf. | We have removed funding information from the manuscript and provide the corrected text in the cover letter. |
| 34. We note that you have indicated that data from this study are available upon request. PLOS only allows data to be available upon request if there are legal or ethical restrictions on sharing data publicly. For more information on unacceptable data access restrictions, please see <http://journals.plos.org/plosone/s/data-availability#loc-unacceptable-data-access-restrictions>.  In your revised cover letter, please address the following prompts:  a) If there are ethical or legal restrictions on sharing a de-identified data set, please explain them in detail (e.g., data contain potentially sensitive information, data are owned by a third-party organization, etc.) and who has imposed them (e.g., an ethics committee). Please also provide contact information for a data access committee, ethics committee, or other institutional body to which data requests may be sent.  b) If there are no restrictions, please upload the minimal anonymized data set necessary to replicate your study findings as either Supporting Information files or to a stable, public repository and provide us with the relevant URLs, DOIs, or accession numbers. For a list of acceptable repositories, please see <http://journals.plos.org/plosone/s/data-availability#loc-recommended-repositories>.  We will update your Data Availability statement on your behalf to reflect the information you provide. | We have expanded the data availability statement, which now gives contact information of the data manager, as well as the ethics committee. |
| 35. Please include captions for your Supporting Information files at the end of your manuscript, and update any in-text citations to match accordingly. Please see our Supporting Information guidelines for more information: <http://journals.plos.org/plosone/s/supporting-information>. | We have included captions for supporting information files at the end of our manuscript. |

**References**

Aaltonen, S., Latvala, A., Rose, R. J., Kujala, U. M., Kaprio, J., & Silventoinen, K. (2016). Leisure-Time Physical Activity and Academic Performance: Cross-Lagged Associations from Adolescence to Young Adulthood. *Scientific Reports, 6*, 39215. <https://doi.org/10.1038/srep39215>

Allison, P. D. (2005). *Fixed Effects Regression Methods for Longitudinal Data Using SAS*. SAS Institute Inc.

Andersen, H. K. (2021). Equivalent approaches to dealing with unobserved heterogeneity in cross-lagged panel models? Investigating the benefits and drawbacks of the latent curve model with structured residuals and the random intercept cross-lagged panel model. *Psychological Methods (forthcoming).* <https://osf.io/hcnfg/download>

Asparouhov, t., & Muthén (2010b). *Bayesian analysis of latent variable models using Mplus.* Muthén & Muthén. <https://www.statmodel.com/download/BayesAdvantages18.pdf>

Asparouhov, T., & Muthén (2010a). *Weighted least squares estimation with missing data.* Muthén & Muthén. [https://www.statmodel.com/download/gstrucmissingrevision.pdf](https://www.statmodel.com/download/GstrucMissingRevision.pdf)

de Looze, M., ter Bogt, T., Hublet, A., Kuntsche, E., Richter, M., Zsiros, E., Godeau, E., & Vollebergh, W. (2013). Trends in educational differences in adolescent daily smoking across Europe, 2002–10. *European Journal of Public Health, 23*(5), 846-852. <https://doi.org/10.1093/eurpub/ckt022>

Hides, L., Chan, G., Dawe, S., McKetin, R., Kavanagh, D. J., Young, R. M., Teesson, M., & Saunders, J. B. (2021). Direction of the relationship between methamphetamine use and positive psychotic symptoms in regular methamphetamine users: evidence from a prospective cohort study. *The British Journal of Psychiatry, 219*(1), 361-367. <https://doi.org/10.1192/bjp.2019.263>

Jang, J. B., Schuler, M. S., Evans-Polce, R. J., & Patrick, M. E. (2019). College attendance type and subsequent alcohol and marijuana use in the U.S. *Drug and Alcohol Dependence, 204*, Article 107580. <https://doi.org/10.1016/j.drugalcdep.2019.107580>

Jindra, C., Li, C., Tsang, R. S. M., Bauermeister, S., & Gallacher, J. (2020). Depression and memory function – evidence from cross-lagged panel models with unit fixed effects in ELSA and HRS. *Psychological Medicine*, 1-9. <https://doi.org/10.1017/S0033291720003037>

Latvala, A., Rose, R. J., Pulkkinen, L., Dick, D. M., Korhonen, T., & Kaprio, J. (2014). Drinking, smoking, and educational achievement: Cross-lagged associations from adolescence to adulthood. *Drug and Alcohol Dependence, 137*, 106-113. <https://doi.org/https://doi.org/10.1016/j.drugalcdep.2014.01.016>

Mackenbach, J. P. (2012). The persistence of health inequalities in modern welfare states: The explanation of a paradox. *Social Science & Medicine, 75*(4), 761-769. <https://doi.org/10.1016/j.socscimed.2012.02.031>

Magnusson Hanson, L. L., Madsen, I. E., Rugulies, R., Peristera, P., Westerlund, H., & Descatha, A. (2017). Temporal relationships between job strain and low-back pain. *Scandinavian Journal of Work, Environment & Health*, 43(5), 396-404. <https://doi.org/10.5271/sjweh.3654>

McKenna, H., Treanor, C., O’Reilly, D., & Donnelly, M. (2018). Evaluation of the psychometric properties of self-reported measures of alcohol consumption: a COSMIN systematic review. *Substance Abuse Treatment, Prevention, And Policy, 13*(1), 6. <https://doi.org/10.1186/s13011-018-0143-8>

Müller, S., Piontek, D., Pabst, A., Baumeister, S. E., & Kraus, L. (2010). Changes in alcohol consumption and beverage preference among adolescents after the introduction of the alcopops tax in Germany. *Addiction, 105*(7), 1205-1213. <https://doi.org/https://doi.org/10.1111/j.1360-0443.2010.02956.x>

Nyberg, A., Peristera, P., Westerlund, H., Johansson, G., & Hanson, L. L. M. (2016). Does job promotion affect men’s and women’s health differently? Dynamic panel models with fixed effects. *International Journal of Epidemiology, 46*(4), 1137-1146. <https://doi.org/10.1093/ije/dyw310>

Peeters, M., Oldehinkel, A., Veenstra, R., & Vollebergh, W. (2019). Unique developmental trajectories of risk behaviors in adolescence and associated outcomes in young adulthood. *PLoS ONE, 14*(11), e0225088. <https://doi.org/10.1371/journal.pone.0225088>

Richter, M., Kuntsche, E., de Looze, M., & Pförtner, T.-K. (2013). Trends in socioeconomic inequalities in adolescent alcohol use in Germany between 1994 and 2006. *International Journal of Public Health, 58*(5), 777-784. <https://doi.org/10.1007/s00038-013-0486-x>

Slutske, W. S. (2005). Alcohol use disorders among us college students and their non–college-attending peers. *Archives of General Psychiatry, 62*(3), 321-327. <https://doi.org/10.1001/archpsyc.62.3.321>

Sweeting, H., Green, M., Benzeval, M., & West, P. J. B. P. H. (2016). The emergence of health inequalities in early adulthood: evidence on timing and mechanisms from a West of Scotland cohort. *BMC Public Health, 16*(1), 41. <https://doi.org/10.1186/s12889-015-2674-5>

Veldman, K., Bültmann, U., Stewart, R. E., Ormel, J., Verhulst, F. C., & Reijneveld, S. A. (2014). Mental health problems and educational attainment in adolescence: 9-year follow-up of the trails study. *PLoS ONE, 9*(7), e101751. <https://doi.org/10.1371/journal.pone.0101751>

Williams, R., Allison, P. D., & Moral-Benito, E. (2018). Linear dynamic panel-data estimation using maximum likelihood and structural equation modeling. *The Stata Journal, 18*(2), 293-326. <https://doi.org/10.1177/1536867X1801800201>

Wu, H. F. (2021). Social determination, health selection or indirect selection? Examining the causal directions between socioeconomic status and obesity in the Chinese adult population. *Social Science & Medicine, 269*, 113564. <https://doi.org/https://doi.org/10.1016/j.socscimed.2020.113564>
