# Supplementary material for: Educational level and alcohol use in adolescence and early adulthood—The role of social causation and health-related selection—The TRAILS Study
Source: PLoS One. 2022 Jan 19;17(1):e0261606. doi: 10.1371/journal.pone.0261606 (PMC8769339; doi:10.1371/journal.pone.0261606)
Supplement: Attachment — Submitted filename: review2_response form.docx [file pone.0261606.s012.docx]

| **Reviewer 1** |  |
| --- | --- |
| Introduction |  |
| The authors may want to also mention additional limitations to the CLPM, namely the convergence effect such that the constructs combine both within and between-person variance. | We would like to thank the reviewer for their helpful comments.  The inability of the traditional CLPM to separate within and between-person variances has now been addressed. We now also state that the FE-CLPM yields within-person associations (p. 6). |
| Methods |  |
| Can you include omega reliability rather than alpha? | We have additionally included McDonald's omega for the effortful control scale (p. 11). Omega could not be computed for the AUDIT-C as the model did not converge even after 100000 iterations. |
| There were significant associations with missing participants and some of the variables. You may want to include a sentence that states you included all these variables in your model to adjust for potential bias due to missing data on these variables. | We have now included a sentence mentioning how we included auxiliary variables (p. 12). |
| I like the inclusion of the sensitivity analyses. You may consider including them in supplemental | The two sensitivity analyses (i.e. removing the binge drinking item from the AUDIT-C, categorical analysis with Bayesian estimator) will be included in the online supplement as S5 and S6 Fig. |
| Results |  |
| The results are organized well. You may consider adding a few subheadings. | Subheadings have now been added to the results section. |
| Do you think the timing of each wave may have impacted the findings? For example, so are spaced further apart in time. | The reviewer highlights an important limitation of our study, which is now discussed in the limitations section (p. 19). |
| **Reviewer 2** |  |
| One final comment relates to the statement on p8: line 201: It is more in line with the cited evidence for this to be written as, "Quantity-frequency measures of alcohol use have shown adequate/good validity and reliability across studies [30]." | We would like to thank the reviewer for this helpful comment.  The sentence now states: “Quantity-frequency measures of alcohol use have shown adequate/good validity and reliability across studies.” (p. 8) |
